# Supplementary material for: Sphingolipid metabolism drives mitochondria remodeling during aging and oxidative stress
Source: bioRxiv. 2025 Feb 27:2025.02.26.640157. Preprint. [Version 1] doi: 10.1101/2025.02.26.640157 (PMC11888424; doi:10.1101/2025.02.26.640157)

bioRxiv preprint doi: https://doi.org/10.1101/2025.02.26.640157; this version posted February 27, 2025. The copyright holder for this preprint (which was not certified by peer review) is the author/funder, who has granted bioRxiv a license to display the preprint in perpetuity. It is made available under aCC-BY 4.0 International license.

Supplementary Figure Legends

Supplemental Figure S1. Characterization of mitochondria morphology changes in chronologically aging yeast cells.

(A) Yeast cells (strain background: BY4741) expressing Tom70-mCherry (red) and Mdh1-GFP (green) were grown to mid-log phase (OD_{600} = 0.5) to initiate chronological aging time course (t=0). Fluorescence microscopy images were collected every 24 hours at the indicated time points.

(B) Mitochondria width was measured (n ≥ 30 cells; ±SD (error bars)) for each time point shown in (A). The indicated p-value was computed using a paired T-TEST comparing the indicated time point to t=0.

(C) Pearson correlation coefficient was computed for individual cells (n ≥ 30 cells; ±SD (error bars)) using Tom70-mCherry (red) and Mdh1-GFP (green) as markers of the outer mitochondrial membrane (OMM) and mitochondrial matrix (MM), respectively. The indicated p-value was computed using a paired T-TEST comparing the indicated time point to t=0.

(D) Yeast cells (strain background: SEY6210) expressing various mitochondrial markers including Om45-mCherry (red), Ach1-GFP (green) and Cit1-GFP (green) were grown to mid-log phase (OD_{600} = 0.5) to initiate chronological aging time course (t=0). Fluorescence microscopy images were collected every 24 hours at the indicated time points.

(E) Yeast cells (strain background: SEY6210) expressing Tom70-mCherry (red) and Mdh1-GFP (green) were grown to mid-log phase (OD_{600} = 0.5) to initiate chronological aging time course (t=0). Cells were then cultured in the indicated growth media and fluorescence microscopy images were collected every 24 hours at the indicated time points.

bioRxiv preprint doi: https://doi.org/10.1101/2025.02.26.640157; this version posted February 27, 2025. The copyright holder for this preprint (which was not certified by peer review) is the author/funder, who has granted bioRxiv a license to display the preprint in perpetuity. It is made available under aCC-BY 4.0 International license.

(F) Mitochondria width was measured (n ≥ 30 cells; ±SD (error bars)) at each time point and condition shown in (F). The indicated p-value was computed using a paired T-TEST comparing the indicated time point to t=0.

(G) Yeast cells (strain background: SEY6210) expressing Tom70-mCherry (red) and Mdh1-GFP (green) and harboring the indicated overexpression plasmid were grown to mid-log phase (OD_{600} = 0.5) to initiate chronological aging time course (t=0). Cells were then cultured in the indicated growth media and fluorescence microscopy images were collected every 24 hours at the indicated time points.

(H) Mitochondria width was measured (n ≥ 30 cells; ±SD (error bars)) at each time point and condition shown in (H). The indicated p-value was computed using a paired T-TEST comparing the t=0 to t=48 for each indicated strain.

(I) Yeast cells (strain background: SEY6210) expressing Tom70-mCherry (red) and Mdh1-GFP (green) and Vph1-mTagBFP2 (blue) were grown to mid-log phase (OD_{600} = 0.5) to initiate chronological aging time course (t=0). Fluorescence microscopy images were collected at the indicated time points.

(J) Intensity of Tom70-mCherry signal in the vacuole lumen (normalized to background) was measured at each of the indicated time points. The indicated p-value was computed using a paired T-TEST comparing the t=0 to each indicated time point.

Supplemental Figure S2. Interventions that modify mitochondrial morphology in chronologically aged yeast cells.

(A) Yeast expressing Tom70-mCherry (red) and Mdh1-GFP (green) were grown to mid-log phase (OD_{600} = 0.5) to initiate chronological aging time course (t=0). Cells were then cultured in SCD media with the indicated glucose concentration and fluorescence microscopy images were collected at the indicated time points.

bioRxiv preprint doi: https://doi.org/10.1101/2025.02.26.640157; this version posted February 27, 2025. The copyright holder for this preprint (which was not certified by peer review) is the author/funder, who has granted bioRxiv a license to display the preprint in perpetuity. It is made available under aCC-BY 4.0 International license.

(B) Mitochondria width was measured (n ≥ 30 cells; ±SD (error bars)) for each condition shown in (A). The indicated p-value was computed using a paired T-TEST comparing the indicated treatment (t=48) to mock treated cells (t=48).

(C) Yeast expressing Tom70-mCherry (red) and Mdh1-GFP (green) were grown to mid-log phase (OD_{600} = 0.5) to initiate chronological aging time course (t=0). Cells were left untreated (mock) or treated with the following compounds associated with rapamycin (rap) or rapamycin + phytosphingosine (rap + PHS) and fluorescence microscopy images were collected at the indicated time points.

(D) Mitochondria width was measured (n ≥ 30 cells; ±SD (error bars)) for each condition shown in (C). The indicated p-value was computed using a paired T-TEST comparing mid-log cells (t=0) to the aged cells (t=48).

Supplemental Figure S3. Sphingolipid-dependent mitochondria remodeling occurs during acute oxidative stress.

(A) Yeast cells (strain background: BY4741) expressing Tom70-mCherry (red) and Mdh1-GFP (green) were grown to mid-log phase (OD_{600} = 0.5) and subject to treatment with the indicated concentration of H_2O_2. Fluorescence microscopy images were collected at the indicated time points following exposure.

(B) Mitochondria width was measured (n ≥ 30 cells; ±SD (error bars)) at each time point. The indicated p-value was computed using a paired T-TEST comparing the indicated time point to t=0.

(C) Pearson correlation coefficient was computed for individual cells (n ≥ 30 cells; ±SD (error bars)) using Tom70-mCherry (red) and Mdh1-GFP (green) as markers of the outer mitochondrial membrane (OMM) and mitochondrial matrix (MM), respectively. The

bioRxiv preprint doi: https://doi.org/10.1101/2025.02.26.640157; this version posted February 27, 2025. The copyright holder for this preprint (which was not certified by peer review) is the author/funder, who has granted bioRxiv a license to display the preprint in perpetuity. It is made available under aCC-BY 4.0 International license.

indicated p-value was computed using a paired T-TEST comparing the indicated time point to t=0.

(D) Yeast cultures were grown to mid-log phase (OD_{600} = 0.5) and subject to treatment with the indicated concentration of H_2O_2 for 12 hours of growth before and plating them on solid YPD media in two-fold serial dilutions.

bioRxiv preprint doi: https://doi.org/10.1101/2025.02.26.640157; this version posted February 27, 2025. The copyright holder for this preprint (which was not certified by peer review) is the author/funder, who has granted bioRxiv a license to display the preprint in perpetuity. It is made available under aCC-BY 4.0 International license.

Supplemental Figure S1

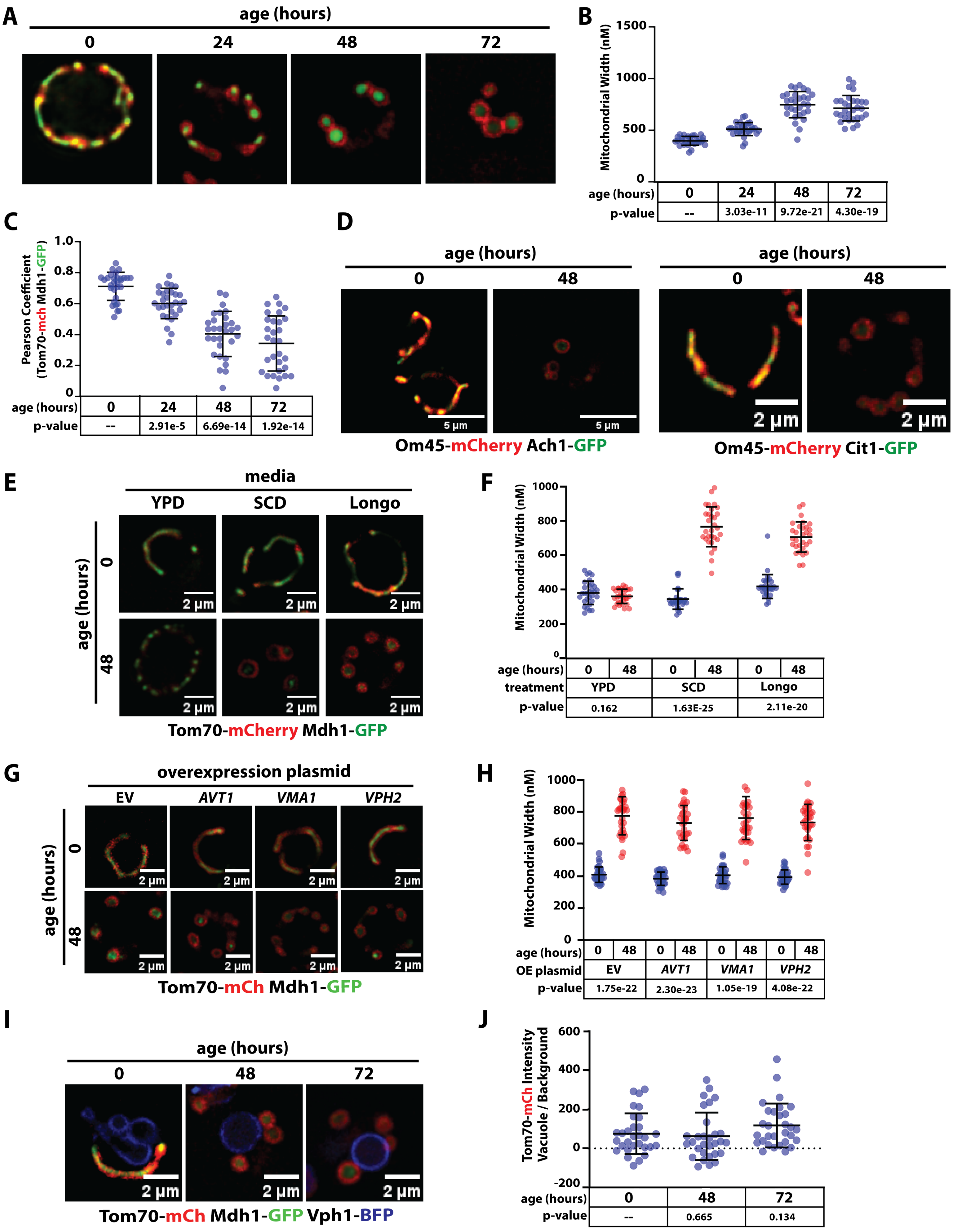

bioRxiv preprint doi: https://doi.org/10.1101/2025.02.26.640157; this version posted February 27, 2025. The copyright holder for this preprint (which was not certified by peer review) is the author/funder, who has granted bioRxiv a license to display the preprint in perpetuity. It is made available under aCC-BY 4.0 International license.

Supplemental Figure S2

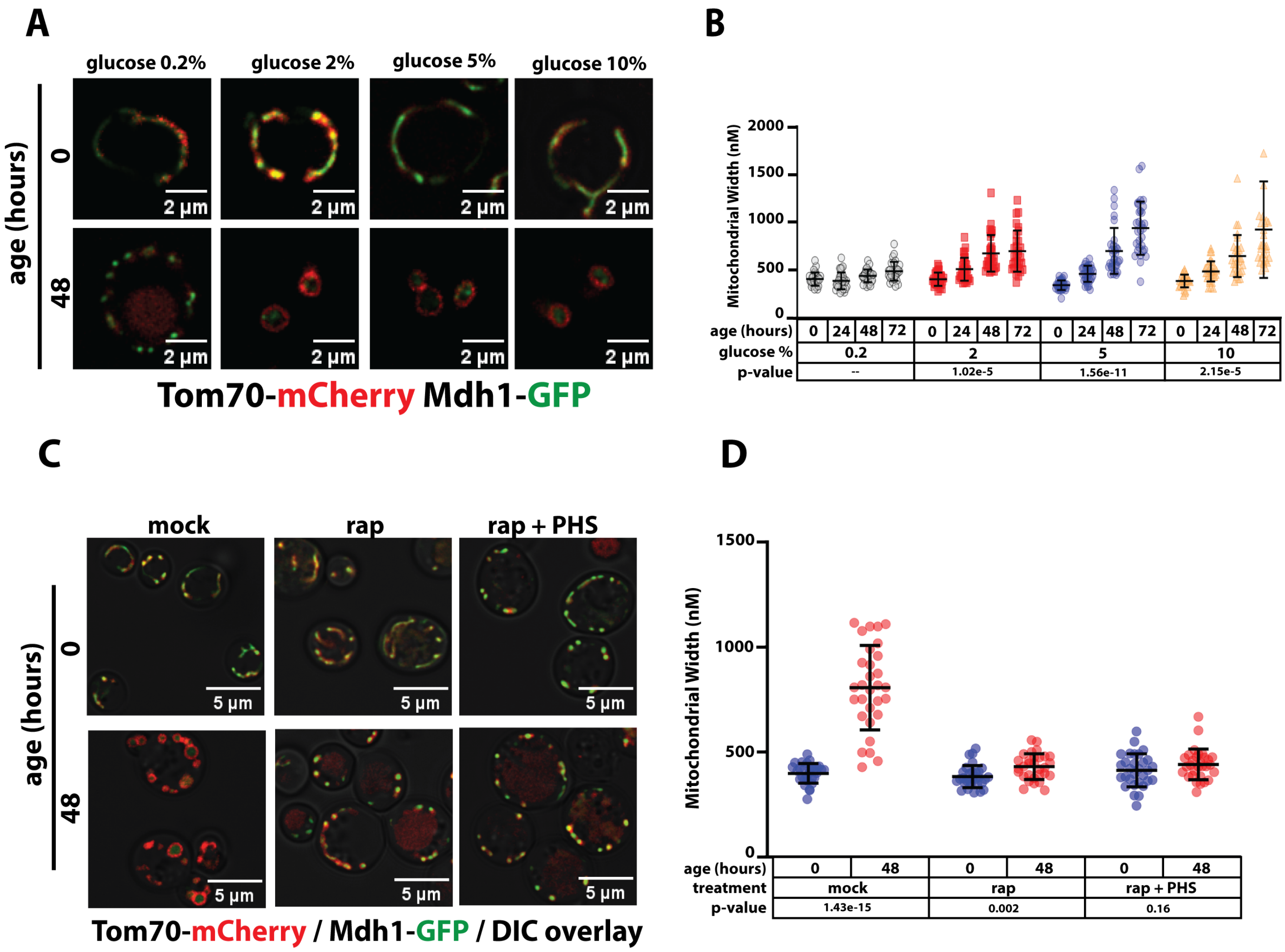

bioRxiv preprint doi: https://doi.org/10.1101/2025.02.26.640157; this version posted February 27, 2025. The copyright holder for this preprint (which was not certified by peer review) is the author/funder, who has granted bioRxiv a license to display the preprint in perpetuity. It is made available under aCC-BY 4.0 International license.

Supplemental Figure S3

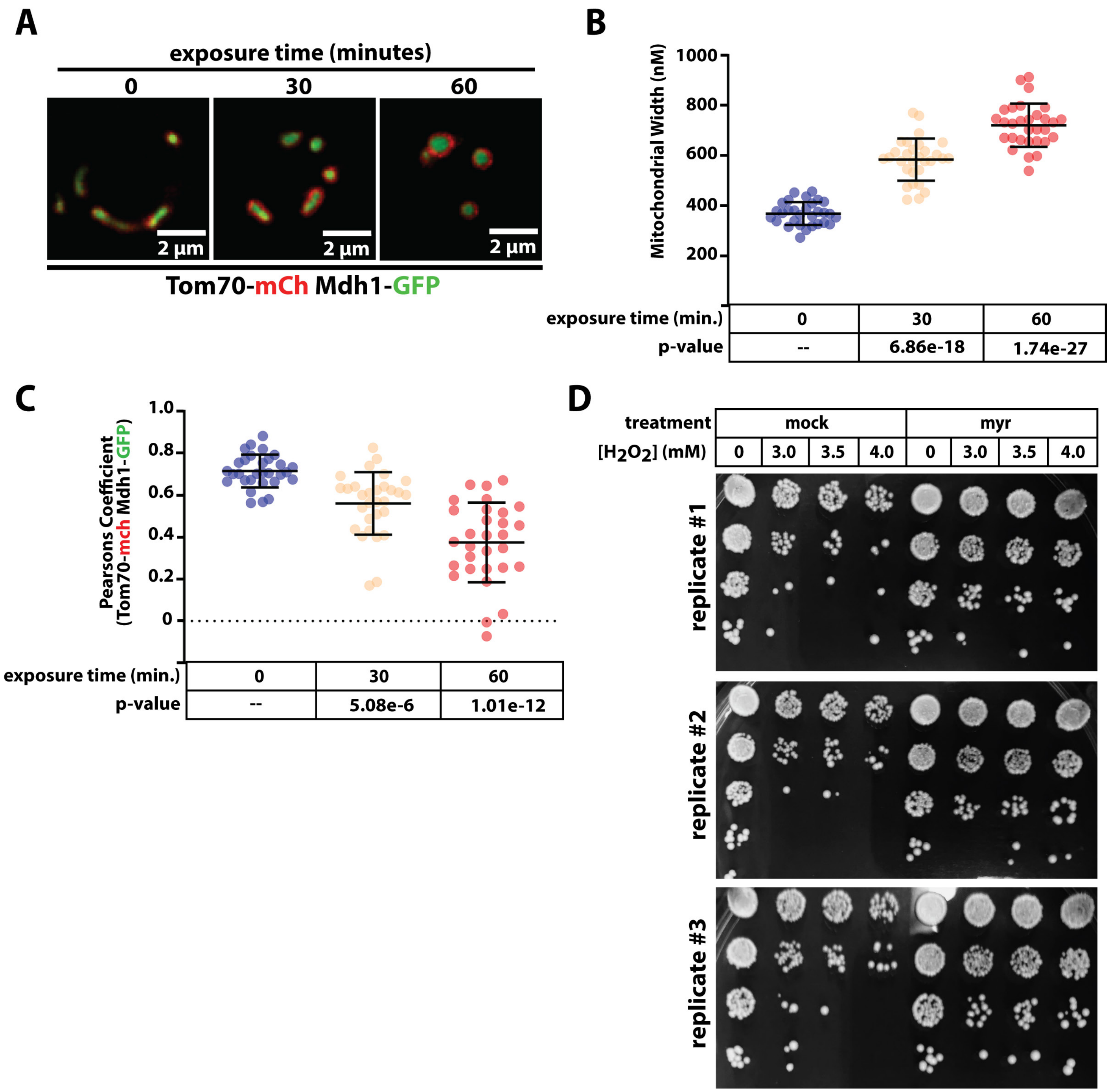

Supplement: 1 [file NIHPP2025.02.26.640157V1-supplement-1.pdf]
